# Hepatoprotective effect of methanol extract of *Agave americana* leaves on paracetamol induced hepatotoxicity in Wistar albino rats

**DOI:** 10.1186/s12906-023-03931-y

**Published:** 2023-04-01

**Authors:** Kassahun Dires Ayenew, Yared Wasihun

**Affiliations:** 1 Department of Pharmacy, Asrat Woldeyes Health Science Campus, Debre Berhan University, Debre Berhan, Ethiopia; 2 Department of Internal Medicine, Ras Desta Damtew Memorial Hospital, Addis Ababa, Ethiopia

**Keywords:** *Agave americana*, Methanol Extract, Hepatoprotective

## Abstract

**Background:**

Ethiopians locally treat liver illnesses with A. Americana. Available literature demonstrates this. However, there are few in-vivo investigations that provide supporting data. The aim of this study was to evaluate the hepatoprotective effects of methanolic extract of *Agave americana* leaves on rat liver damage caused by paracetamol.

**Methods:**

The acute oral toxicity test was conducted in accordance with OECD-425 recommendations. The approach outlined by Eesha et al. (Asian Pac J Trop Biomed 4:466-469,  2011) was used to test the hepatoprotective activity. Wistar male rats weighing between 180 and 200 g were used, and six groups with seven animals each were formed. Group I received treatment with gum acacia (2%) at a dose of 2 ml/kg p.o. daily for 7 days. Rats in group II were treated with 2% gum acacia orally daily for seven days along with a single dose of paracetamol (2 mg/kg) p.o. on 7^th^ day. Silymarin (50 mg/kg) was given orally to Group III for 7 days. Plant extract doses of 100 mg/kg, 200 mg/kg, and 400 mg/kg were administered orally to Groups IV -VI for seven days, respectively. All rats in groups III-VI were treated with paracetamol (2 mg/kg) 30 min following extract administration. Blood samples were obtained from the cardiac puncture after paracetamol had been used for 24 h to induce toxicity. Serum biomarkers (AST, ALT, ALP, and total bilirubin) were estimated. A histopathological investigation was also done.

**Results:**

No toxicity symptoms or animal fatalities were recorded during the acute toxicity study. The values of AST, ALT, ALP, and total bilirubin were all substantially raised by paracetamol. Significant hepatoprotective effects were obtained by pretreatment with *A. americana* extract. Histopathological examination of the liver tissues of paracetamol control group represented the presence of marked foci of mononuclear infiltration in the hepatic parenchyma tissue, sinusoid, and around central vein, as well as disorganization of hepatic plates, necrosis, and fatty changes of hepatocytes. Pretreatment with *A. americana* extract reversed these alterations. Results of the methanolic extract of *A. americana* were comparable to Silymarin.

**Conclusion:**

The current investigation supports the hepatoprotective properties of *Agave americana* methanolic extract.

## Introduction

The liver, which plays a key role in metabolism, detoxification, and excretion of different endogenous and exogenous chemicals, is the most significant organ in the human body. The liver produces highly reactive molecules known as free radicals. Damage to tissue results when free radicals form a covalent link with cell membrane lipids, changing the permeability of the cell membrane [1].

Around 10% of the world's population suffers from liver ailments. This comprises cirrhosis, fibrosis, chronic hepatitis, alcoholic steatosis, and hepatocellular cancer [2]. Liver disease morbidity and mortality, particularly in poorer nations, is a significant public health issue on a global scale. Modern medicine still faces difficulties in treating liver illness. Only corticosteroids and immunosuppressive medications are approved to treat liver problems. These, however, have several negative side effects. Increased reliance on complementary and alternative medicine, particularly herbal treatment, has resulted from this. It is well recognized that plant medicines are essential for treating liver conditions [3].

As a non-narcotic analgesic and antipyretic, paracetamol (acetaminophen) is often used. When consumed in deadly quantities, it transforms into a powerful hepatoxin that causes fulminated hepatic and renal tubular necrosis, which is fatal to humans and many other species of animals including rats [4]. Acute neuroinflammatory liver diseases with large elevations in serum SGPT and SGOT levels are like the laboratory characteristics of acetaminophen-induced hepatotoxicity. The histological findings of the liver biopsy or autopsy demonstrated a variable degree of centrizonal necrosis without steatosis and with a relatively mild inflammatory infiltrate [5].

The primary metabolic pathways for paracetamol are sulphation and glucuronidation, which produce unreactive metabolites. The cytochrome P-450 system subsequently activates these unreactive metabolites to cause liver damage [6]. A drug's electrophilic metabolite appears to be what causes acetaminophen's recognizable zone 3 necrosis (N-acetyl-p-benzoquinonimine, NAPQI). NAPQI is first detoxified by forming mercapturic acid by conjugation with reduced glutathione [7]. However, NAPQI will oxidize tissue macromolecules, such as lipids or protein thiols, and change the homeostasis of calcium after depleting glutathione, leading to cell death. This occurs when the rate of NAPQI synthesis surpasses the rate of detoxication by glutathione.

The damaging mechanism in liver damage brought on by acetaminophen intake has been theorized to be lipid peroxidation [8]. Following acetaminophen treatment, liver tissue protective effects coincided with antioxidant activity, indicating that lipid peroxidation and free radical production may both contribute to this kind of drug damage mechanism.

In the developing world, herbal medications are increasingly in high demand for basic healthcare not because they are cheap but rather because they have few adverse effects and are readily available in nature [9].

200–300 different species of *Agave americana* may be found worldwide, primarily in tropical and temperate climates. The family is represented by 6 genera and 20 species in Ethiopia. It may be found in East Africa from sea level to 2500 m above sea level. Both regions with low and high rainfall rates contain it. It is regarded as an invasive noxious weed in several southern African nations, notably South Africa [10].

According to reports, *A. americana* Linn. (Family: *Agavaceae*) leaves can be used as a hepatoprotective, antimicrobial, and to treat a variety of liver conditions [11, 12]. The antioxidant activity of *A. americana* extract has been studied in the past [13] and this research raises the prospect of employing the plant as a hepatoprotective agent.

Despite of the popular use of *A. americana* as a medicinal plant, there is a lack for adequate in vivo data on its hepatoprotective activity. The aim of this study was to evaluate the hepatoprotective effect of *A. americana* against paracetamol induced hepatotoxicity in rats.

## Materials and methods

### Experimental animals

A total of 42 healthy Male Wistar albino rats, weighing between 180–200 g were obtained from Ethiopian Public Health Institute (EPHI) and used to determine the hepatoprotective activity. The animals were housed in clean plastic cages and maintained under standard conditions of temperature (24 + -2) °C under 12 h light/dark cycle. They were fed with standard pellet diet and water ad libitum. All the animals were acclimatized to laboratory conditions before commencement of the experiment. The procedure followed in this study was done in accordance with Animals Ethics Committee guidelines [14].

### Collection and preparation of methanolic extract of *A. americana*

Fresh *A. americana* leaves were collected from a wild source in Addis Ababa's Yeka sub-city after receiving a permission from Ethiopian Biodiversity Conservation Institute, Addis Ababa, Ethiopia. Collection of the plant materials complies with relevant national and international guidelines and legislations. Professor Ensermu Kelbessa of Addis Ababa University in Ethiopia assisted in the identification and authentication of the plant. The specimen has been stored in Addis Ababa University herbarium under voucher 084,908. The plant material that had been dried and ground into a powder was macerated in methanol (80%) at a 1:1 ratio. After that, filter paper was used to clean it (Whatman No 3, Whatman Ltd., England). Then, the filtrated extract was concentrated by rota vapor (Buchii model R-200, Switzerland) at a temperature of 40 ^0^C. The aqueous residue was then heated to 40 ^0^C in an oven for approximately 48 h. The powder obtained was kept in desiccators using amber-colored glass bottles [15].

### Preliminary phytochemical screening

The preliminary phytochemical screening was carried out using qualitative chemical techniques. The methanolic extract of *A. americana* was analyzed for the presence of sugars, alkaloids, triterpenoids, saponins, phenols, sterols, and flavonoids [16].

### Acute toxicity study

According to OECD-425 recommendations, the acute oral toxicity test was conducted. The dose of 5000 mg/kg was given to five male Wistar albino rats. Rats were continually observed for obvious behavioral changes for the first four hours following administration of *A. americana* extract, and then the observation was continued at regular intervals for the next 24 and 72 h for a total of 14 days. All the animals successfully tolerated the corresponding dose with no evidence of toxicity or death. The oral LD50 exceeded 5000 mg/kg. Three separate graded doses of 100 mg/kg, 200 mg/kg, and 400 mg/kg were selected to evaluate the hepatoprotective effect [17].

### Hepatoprotective activity

The approach outlined by Eesha et al., 2011 [18] was used to test the hepatoprotective activity. Wistar male rats weighing between 180 and 200 g were used, and six groups with seven animals each were formed. Group I received treatment with gum acacia (2%) at a dose of 2 ml/kg p.o. daily for 7 days. Rats in group II were treated with 2% gum acacia orally daily for seven days along with a single dose of paracetamol (2 mg/kg) p.o. on 7^th^ day. Silymarin (50 mg/kg) was given orally to Group III for 7 days. Plant extract doses of 100 mg/kg, 200 mg/kg, and 400 mg/kg were administered orally to Groups IV -VI for seven days, respectively. All rats in groups III—VI were treated with paracetamol (2 mg/kg) 30 min following extract administration. Paracetamol had been used for 24 h to induce toxicity and then blood sample from each mouse was collected separately in sterilized dry centrifuge tubes by cardiac puncture and allowed to coagulate for 10 min at 37 ^**0**^C. The clear serum was separated at 2500 rpm for 10 min and subjected to biochemical estimations like aspartate aminotransferase (AST), alanine aminotransferase (ALT), alkaline phosphate (ALP), and total bilirubin using diagnostic kits [19].

### Serum biochemical analysis

Liver biochemical tests (AST, ALT, and ALP) were performed on a clinical chemistry automatic analyzer (ADVIA 2400, Bayer Diagnostics). AST, ALT, and ALP were measured according to the previous methods [20, 21] using commercial assay kit (Bayer Diagnostics). The serum total concentrations of bilirubin were measured adhering to the scientific methods [22, 23] utilizing commercial kits on the Express Plus biochemical analyzer (Ciba-Corning Diagnostics).

### Histopathological studies

The rats were scarified through sodium pentobarbital after the blood sample was taken, and the liver was then removed from the animals and rinsed with normal saline. The absolute weight of the liver tissue was measured. The tissues were treated individually and fixed in 10% formalin for histological analysis. Using a microscope, a pathologist who was unaware of the study's procedure inspected the microscopic slides [24].

### Statistical analysis

Means and standard errors of the means (SEM) were used to express the results. One-way analysis of variance (ANOVA) was used to analyze the data. Statistics were deemed significant at *P* < O.05. The SPSS application (version 21.0) was utilized to perform the statistical analysis.

## Results

### Preliminary phytochemical analysis

Preliminary phytochemical analysis showed the presence of phytoconstituents such as flavonoids, tannins, saponins and alkaloids.

### Acute toxicity study

During the 14-day study period up to the dose of 5000 mg/kg body weight p.o. for the methanolic extract of *A. americana* leaves, there were no reported side effects or animal deaths. Therefore, three doses of the extract were chosen for testing the hepatoprotective action against paracetamol-induced toxicity: 100 mg/kg, 200 mg/kg, and 400 mg/kg body weight P.O [17].

### Effect of methanol extract of *A. americana* on AST, ALT, ALP, and total bilirubin

Wistar rats treated with paracetamol (2 mg/kg p.o.) alone developed significant hepatocellular damage, as evidenced by an increase in serum biomarkers AST, ALT, ALP, and total bilirubin when compared to the control group, according to the findings of the hepatoprotective study of the methanolic extract of *A. americana*. Prior to the administration of paracetamol, rats were pretreated with a methanolic extract of *A. americana* leaves at doses of 100 mg/kg, 200 mg/kg, and 400 mg/kg. This resulted in a substantial decrease in the levels of AST, ALT, ALP, and total bilirubin that was nearly equivalent to Silymarin. When administered at a dose of 400 mg/kg compared to 100 mg/kg, the methanolic extract of *A. americana* demonstrated a dose-dependent action, as indicated by the lowered levels of blood enzymes and total bilirubin (Table 1).

**Table 1:** Effect of methanol extract of *A. americana* on biochemical parameters in paracetamol intoxicated rats

**Groups**	ALT (U/L)	AST (U/L)	ALP (U/L)	Total Bilirubin (mg/dl)
Control	28.31 ± 2.51	31.27 ± 1.24	194.00 ± 6.60	0.50 ± 0.06
Paracetamol control	20.29 ± 2.21*	187.60 ± 2.83*	617.10 ± 9.51*	3.35 ± 0.07*
Silymarin	33.50 ± 1.61**	36.40 ± 1.05**	270.50 ± 3.14**	0.61 ± 0.03**
100 mg/kg A. americana	42.75 ± 0.99**	45.08 ± 1.04**	297.90 ± 8.30**	1.42 ± 0.05**
200 mg/kg *A. americana*	38.18 ± 0.75**	42.43 ± 1.01**	283.90 ± 4.11**	0.81 ± 0.05**
400 mg/kg A. americana	36.10 ± 2.38**	38.32 ± 0.77**	273.00 ± 1.01**	0.70 ± 0.05**

Data were expressed as mean ± SEM (*n* = 6), *P < 0.05 compared with control, **P < 0.05 compared with paracetamol

### Effect of methanol extract of *A. americana* on histopathology

**Fig. 1:**
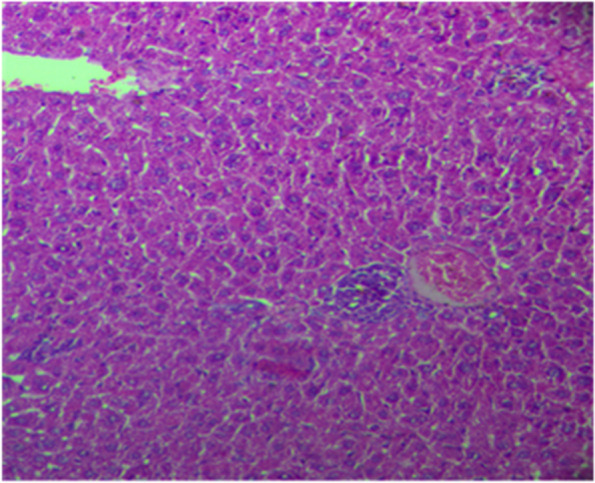
Section of liver from the control group showing normal architecture of the hepatic lobule with no histopathological change H&E staining (× 100)

**Fig.2:**
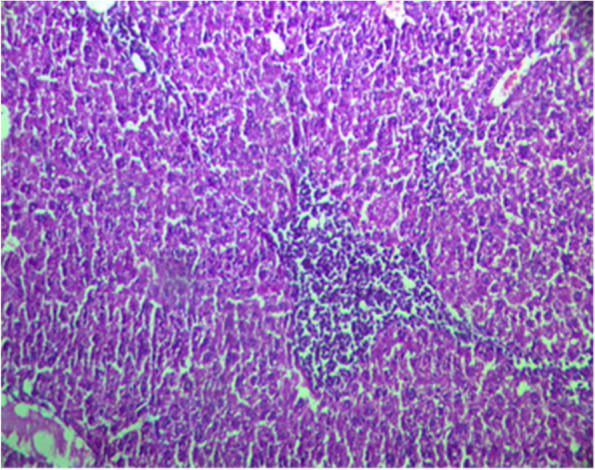
Liver section from rat treated with Paracetamol at a dose of 2 mg/kg; b. w showing a marked foci of mononuclear infiltration H&E staining (× 100)

**Fig. 3:**
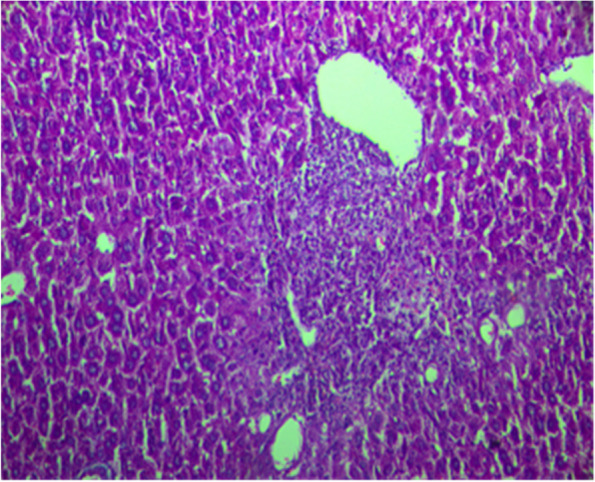
Liver section from rat treated with Silymarin and Paracetamol showing normal architecture of the hepatic lobule with no histopathological change H&E staining (× 100)

Histological analysis of the liver confirmed the outcomes of blood enzyme testing and demonstrated the hepatoprotective properties of the methanolic extract of *A. americana* leaves. The typical control is depicted in Fig. 1. Rats given paracetamol alone displayed piecemeal liver necrosis, along with distinct areas of mononuclear infiltration in the hepatic parenchyma tissue, sinusoid, and around the central vein. The liver also displayed disorganized hepatic plates, necrosis, and fatty alterations in hepatocytes (Fig. 2). As seen in Fig. 3, the Silymarin-treated group preserved the normal morphology and recovered blood marker and bilirubin levels equivalent to methanolic *A. americana* treated groups.

**Fig. 4:**
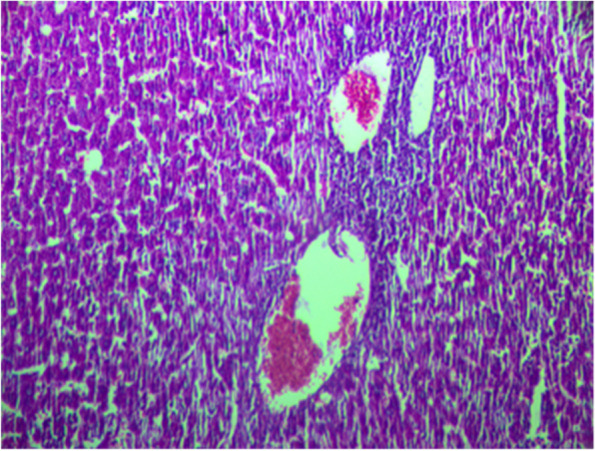
Liver section from rat treated with 100 mg/kg methanol extract of *Agave americana* and Paracetamol showing normal architecture of the hepatic lobule with minimal focus of mononuclear infiltration at the periphery (arrow) H&E staining (× 100)

**Fig. 5:**
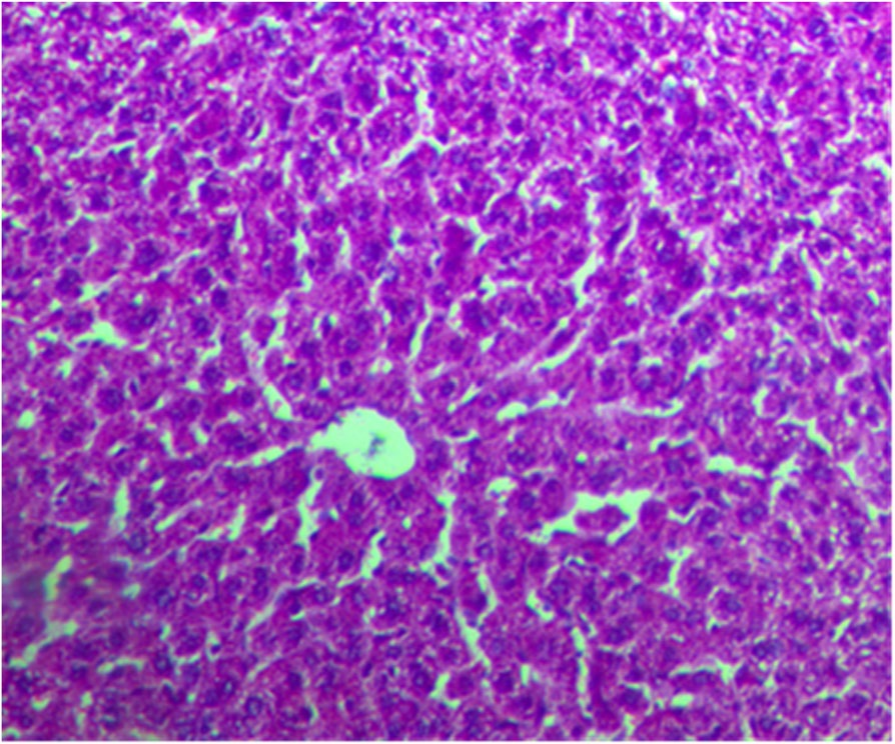
Liver section from rat treated with 200 mg/kg methanol extract of *Agave americana* and Paracetamol showing normal architecture of the hepatic lobule with no histopathological change H&E staining (× 100)

**Fig. 6:**
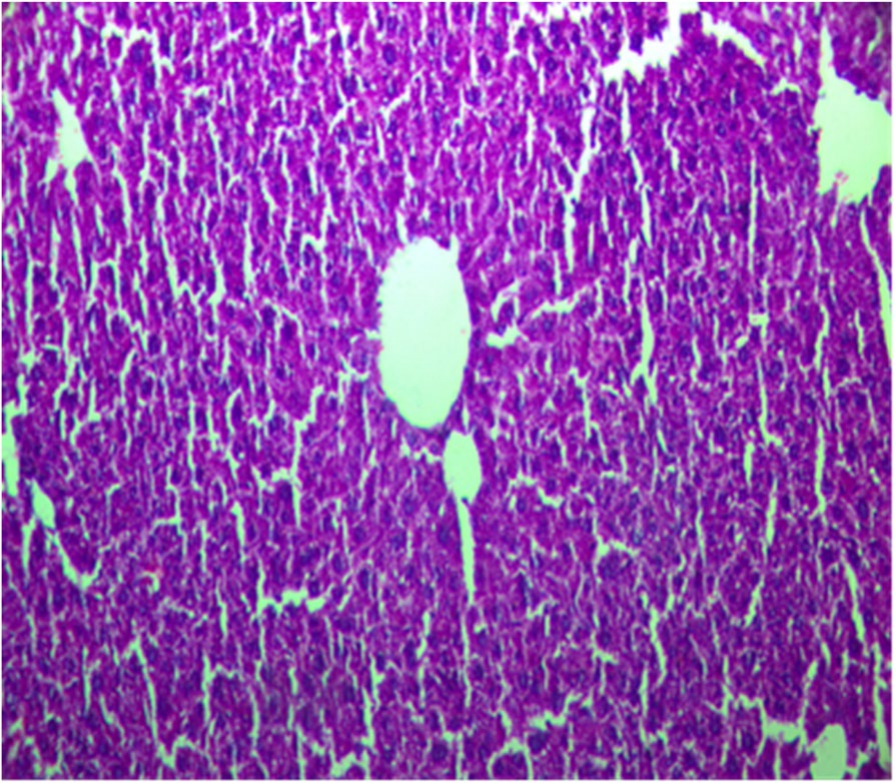
Liver section from rat treated with 400 mg/kg methanol extract of *Agave americana* and Paracetamol showing normal architecture of the hepatic lobule with no histopathological change H&E staining (× 100)

In comparison to the standard group, the pretreated methanolic extract groups IV, V, and VI had normal liver histology and well characterized hepatic architecture (Figs. 4, 5 and 6).

## Discussion

As significant sources of bioactive compounds with positive effects on health, medicinal plants are receiving a lot of attention. The question of safety and toxicity, however, places significant restrictions on the usage of therapeutic herbs. A common sign of the toxicity of medicinal herbs in vivo is liver injury [25, 26].

According to David et al., 2014 [27], the blood biomarkers of liver function include the aminotransferases (ALT, AST), ALP, and bilirubin, with an increase in these markers suggesting hepatic damage. In contrast, total protein and albumin levels are lowered in the presence of hepatic damage [28].

The little change in blood levels of ALT, AST, and ALP, as well as total bilirubin in liver damage, show that pretreatment of normal rats with *A. americana* had no harmful or negative consequences. The current study's findings supported the hypothesis that paracetamol has hepatotoxic effects since it significantly increased the activity of the liver function marker enzymes ALT, AST, ALP, and bilirubin in the serum of rats. These findings are consistent with related researches [29–31].

The increased levels of ALT, AST, ALP, and bilirubin in paracetamol-treated rats were dramatically lowered by *A. americana* pretreatment. The stabilizing effect of the *A. americana* phytochemical constituent(s) and various active ingredients on the plasma membrane of the hepatocytes, likely caused by the stimulation of hepatocellular protein synthesis and ability to induce microsomal enzymes either by accelerating the excretion of paracetamol or by inhibiting oxidative stress induced by paracetamol, may be responsible for the decreased levels of these serum biomarkers [32, 33].

AST and ALT are most frequently linked to liver parenchyma cells. Acute liver injury is characterized by elevated AST and ALT values [34]. Furthermore, intrahepatic cholestasis and infiltrative liver disorders cause an increase in ALP levels [35].

Large amounts of enzymes leaking into the circulation are linked to liver centrilobular necrosis [36]. In the current investigation, administration of *A. americana* extract resulted in the levels of these enzymes returning to normal range, showing the plant's hepatoprotective activity. Preserving the typical physiological processes of the hepatic organs that have been disrupted by hepatotoxins is a solid criterion for evaluating the effectiveness of any hepatoprotective therapy. Similar reports were observed from some other plant species including *Aerva lanata* [37] and *Red Lentil* [38].

The activity of hepatic cells was correlated with blood bilirubin levels [39]. When treated with hepatotoxins (paracetamol), high blood bilirubin content indicates that the liver damage causing a high rate of erythrocyte breakdown [40].

In this investigation, the plant extract resulted in the amount of bilirubin returning to normal levels, suggesting the plant's hepatoprotective activity. The effect was shown to be equivalent to common medications (Silymarin). Results generally imply that the protective action of *A. americana* extract normalizes the unbalanced antioxidant system in liver treated with paracetamol. Histopathological examination was used to further examine the hepatoprotective effect of *A. americana* extract.

The results of serum biochemical investigations and the histopathological analysis of liver samples were consistent, showing that *A. americana* extract can prevent the hepatotoxicity that paracetamol causes. Since phenolics and flavonoids often scavenge free radicals and play a crucial part in reducing oxidative stress, they exhibit a wide spectrum of biological and pharmacological activities [41].

Pretreatment with *A. americana* extract protected hepatic architecture and liver tissue from marked foci of mononuclear infiltration of hepatic parenchyma tissue and sinusoid. Similar reports from a few other plants, such as *Mung bean* [42], *Deinococcus radiodurans* [43] and *Lumnitzera racemosa *[44] were also noted.

Previous studies have shown that paracetamol induces apoptosis and necrosis in addition to fibrosis, mononuclear cell infiltration, steatosis, and the degradation of hepatocytes in the liver, all of which are histopathological alterations indicative of liver injury [44]. Thus, the histological results in the liver caused by paracetamol are consistent with earlier research.

Hepatoprotective drugs may aid in the process of regeneration, the prevention of fibrosis, or the development of nodules that may manifest after long-term use [45]. This study revealed distinct areas of mononuclear infiltration in the sinusoid and around the central vein, as well as the disorganization of the hepatic plates, necrosis, and fatty changes in the hepatocytes caused by the hepatotoxic drug paracetamol. The inhibition of these changes return to normal by *A. americana* extract.

Previous studies indicated the anti-inflammatory and antioxidant activities of *A. Americana* leave extracts [46, 47] which suggests the hepatoprotective potential of this plant in the current investigation. Moreover, Flavonoids, tannins, saponins, and alkaloids were found in the methanolic extract of *A. americana*, according to a preliminary phytochemical investigation. It is known that these phytochemicals have hepatoprotective properties [48, 49]. Pretreatment with *A. americana* extract shielded hepatic architecture and liver tissue from pronounced foci of mononuclear infiltration of hepatic parenchyma tissue, sinusoid, and around central vein, as well as from tissue disorganization and necrosis, by preventing the toxic chemical reaction, oxidative stress, and molecular changes in the liver tissues that ultimately cause necrosis.

Histopathological examination of liver from paracetamol intoxicated rat pretreated with *A. americana* revealed enhanced hepatocellular architecture, which indicates the hepatoprotective effects of the plant.

## Conclusions

*A. americana* leaf methanolic extract has hepatoprotective action against paracetamol-induced hepatotoxicity in rats, according to the findings of this study. Regarding the quantitative phytochemical screening and mechanism of action at molecular level, future investigations could be planned. To determine if it is safe for people, studies on long-term toxicity should be employed, and more researches are needed to verify the current findings.

## Data Availability

This document contains most of the data. Upon reasonable request, Kassahun Dires Ayenew, the author of this manuscript, will provide more information. Email: kassh2009@gmail.com.

## Abbreviations

AEC: Animal Ethics Committee
ALT: Alanine Transaminase
ALP: Alkaline Phosphatase
AST: Aspartate Transaminase
LD_50_: Median Lethal dose
OECD: Organization for Economic Cooperation and Development
TB: Total bilirubin
